# Nonconsumptive effects in a multiple predator system reduce the foraging efficiency of a keystone predator

**DOI:** 10.1002/ece3.691

**Published:** 2013-08-01

**Authors:** Jon M Davenport, David R Chalcraft

**Affiliations:** Division of Biological Sciences, University of MontanaMissoula, Montana, 59812

**Keywords:** Amphibian, consumptive effects, food web, keystone predator, multiple predator, multiplicative risk model, nonconsumptive effects, temporary ponds

## Abstract

Many studies have demonstrated that the nonconsumptive effect (NCE) of predators on prey traits can alter prey demographics in ways that are just as strong as the consumptive effect (CE) of predators. Less well studied, however, is how the CE and NCE of multiple predator species can interact to influence the combined effect of multiple predators on prey mortality. We examined the extent to which the NCE of one predator altered the CE of another predator on a shared prey and evaluated whether we can better predict the combined impact of multiple predators on prey when accounting for this influence. We conducted a set of experiments with larval dragonflies, adult newts (a known keystone predator), and their tadpole prey. We quantified the CE and NCE of each predator, the extent to which NCEs from one predator alters the CE of the second predator, and the combined effect of both predators on prey mortality. We then compared the combined effect of both predators on prey mortality to four predictive models. Dragonflies caused more tadpoles to hide under leaf litter (a NCE), where newts spend less time foraging, which reduced the foraging success (CE) of newts. Newts altered tadpole behavior but not in a way that altered the foraging success of dragonflies. Our study suggests that we can better predict the combined effect of multiple predators on prey when we incorporate the influence of interactions between the CE and NCE of multiple predators into a predictive model. In our case, the threat of predation to prey by one predator reduced the foraging efficiency of a keystone predator. Consequently, the ability of a predator to fill a keystone role could be compromised by the presence of other predators.

## Introduction

Predators exert their influence on prey via a consumptive effect (CE) that kills prey and a nonconsumptive effect (NCE) that causes prey to alter their behavior, life history, or morphology (Sih et al. 1985; Abrams 1995). Although the importance of a predator's CE is well known, recent studies reveal that the NCE of predators on prey demographics can also be important, and in some cases more important (Werner and Peacor 2003; Schmitz et al. 2004; Preisser et al. 2005). Furthermore, the NCE of predators on some prey traits (e.g., reduced foraging activity) can alter the mortality risk of prey (i.e., the CE) to predators (Werner and Peacor 2003; Peckarsky et al. 2008). Consequently, much effort has focused on understanding factors mediating the intensity of NCEs (Preisser et al. 2007, 2009; Preisser and Bolnick 2008). Less well studied is the issue of how the CE and NCE of multiple predator species interact to influence the combined effect of a predator assemblage on prey mortality.

Understanding how the CE and NCE of different predators interact to determine the combined CE of multiple predators is essential because most prey in nature are exposed to multiple predator species (Polis 1991). Furthermore, the combined CE of multiple predators on their prey can differ from that expected if predators affect prey independently of each other (Sih et al. 1998; Vance-Chalcraft et al. 2004). A difference between the observed and expected CE of multiple predator species is referred to as an emergent multiple predator effect (MPE). The occurrence of an emergent MPE is a consequence of one predator species altering the CE of another predator species on a shared prey. Such alterations in the CE of one predator on its prey can be due to the NCE of other predators on each other or on their shared prey. NCEs that influence the occurrence of an emergent MPE could derive from (1) direct physical interactions among the predators (e.g., interference competition) or between predators and their prey (e.g., predators actively chasing prey into the jaws of other predators) or (2) nonphysical interactions, such as chemical signals, alerting prey to the presence of a predator (Soluk and Collins 1988; Werner and Peacor 2003). Ample evidence exists to demonstrate that prey alter their behavior, morphology, and/or life history as a result of nonphysical interactions with their predators (Lima and Dill 1990; Tollrian and Harvell 1999), but it remains unknown whether these responses alter the CE of different predators in a way that adequately explains the occurrence of an emergent MPE.

Although some studies have observed mechanisms involving NCEs of predators that could explain the occurrence of an emergent MPE (e.g., Soluk and Collins 1988; Soluk 1993; Sokol-Hessner and Schmitz 2002; Schmitz 2007), their experimental designs have not afforded the opportunity to evaluate whether the proposed NCE is sufficient to explain the emergent MPE. The observation that a particular mechanism is occurring does not necessarily mean that it is sufficient to explain an emergent MPE. For example, a documented change in prey behavior by one predator (i.e., a NCE) may be consistent with one explanation of an emergent MPE, but this information alone does not necessarily mean that one can better predict the combined impact of the multiple predators. To do this, one needs to parameterize a model that accounts for the proposed mechanism (i.e., the NCEs) contributing to the emergent MPE and evaluate whether this model adequately predicts the combined effect of the multiple predator species. Others have attempted to examine the extent to which different mechanisms contribute to an emergent MPE (Relyea and Yurewicz 2002; Crumrine and Crowley 2003; Griffen and Byers 2006; Rudolf 2008), but these studies have either lacked the necessary treatments to distinguish between NCE produced via physical and nonphysical mechanisms, involved intraguild predation, or employed an inappropriate model for evaluating the occurrence of an emergent MPE (Appendix S1).

We examined the potential for an emergent MPE on prey mortality to arise via NCEs in a multiple predator system involving the known keystone predator, *Notophthalmus viridescens* (newts hereafter) (Morin 1983; Wilbur 1987; Chalcraft and Resetarits 2003a), larval *Anax* (dragonflies), and larval toads (*Bufo* hereafter). Although Wilbur and Fauth (1990) also examined the independent and interactive effects of these same predators on *Bufo* tadpoles, they tested whether the combined CE of both predators statistically differed from that expected from an additive model of predation risk. However, the most appropriate model to generate expected responses of prey to multiple predators when prey are unable to reproduce or immigrate into the system (the conditions prey experienced in Wilbur and Fauth's study) is the multiplicative risk model (Sih et al. 1998). Although Wilbur and Fauth (1990) distinguish between the multiplicative and additive risk models they statistically compared their observations to that predicted by the additive model (Sih et al. 1998). Furthermore, Wilbur and Fauth (1990) did not assess the NCEs of predators. We conducted two experiments to examine the interaction between the CE and NCE of different predators on their prey in order to advance our understanding of how well we can predict the combined CE of multiple predators on their prey.

## Materials and Methods

Our first experiment was a short-term experiment designed to assess the CEs and NCEs of multiple predator species on a shared prey resource. Our experimental design allowed us to evaluate the extent to which NCEs produced by nonphysical interactions affects the combined CE of multiple predator species on their prey. The second experiment allowed us to assess the NCE of predators on prey in order to identify the mechanism contributing toward an emergent MPE found in the first experiment. We focused on NCEs of predators as manifested through prey behavior because the duration of the first experiment was sufficiently short that only behavioral changes, and not changes in morphology or life history, could result in the production of an emergent MPE. Appendix S2 describes where and when study animals were collected.

### Experiment 1

We assessed the CE of multiple predator species and the extent to which NCE contribute toward the production of an emergent MPE by conducting an experiment in plastic tubs (58.4 × 42.5 × 15.2 cm). Tubs were filled with 13 L of filtered water and stocked with 150 g of washed sweet gum (*Liquidambar styraciflua*) leaves collected from a natural pond. Each tub received 30 newly hatched *Bufo* tadpoles (Gosner stages 23–28). We independently manipulated the abundance (zero vs. one) of two free-swimming predator species (newts and *Anax*) as is typical of studies examining CEs of predators and emergent MPEs. The average mass (±SE) of adult newts in the experiment was 1.36 g ± 0.06 and the average mass of the late-instar *Anax* larvae in the experiment was 1.14 g + 0.07. Densities of *Bufo* tadpoles (125/m^2^), larval *Anax* (4/m^2^), and adult newts (4/m^2^) are reflective of densities found in natural ponds (Appendix S2).

Our study included two additional treatments that are not typically included in studies examining MPE; one where an *Anax* could consume *Bufo* in the presence of a caged newt and one where a newt could consume *Bufo* in the presence of a caged *Anax*. These treatments allowed us to measure the extent to which one predator species alters the foraging rate of a second predator species without physically interacting with the other predator or their prey. Cages for predators were 0.27 L opaque plastic cups with pin holes punched in the side which provided an opportunity for predators and prey to sense the presence of another predator species via chemical cues while preventing physical interactions or visual detection. All tubs received a cage so as to not confound the occurrence of a caged predator with the occurrence of a cage.

Treatments were randomly assigned to tubs and predator individuals were randomly assigned to tubs of the appropriate treatment. Prey were added to tubs 2 h before predators. We emptied tubs and counted the number of surviving *Bufo* 24 h after the addition of predators. Each of the six treatments was replicated once within each of seven blocks (see Appendix S2 for description of blocking structure).

We estimated instantaneous mortality rates (# tadpoles that die/individual/24 h) of *Bufo* in each tub as the absolute value of the ln proportion of *Bufo* that survived to the end of the experiment. We used the absolute value so that mortality risk is represented by a positive value. This approach assumes that neither reproduction nor migration occurs and that the number of *Bufo* present declines in an exponential manner (Gotelli 2008); all of which are reasonable assumptions in our study. We performed analyses on instantaneous mortality rates because the multiplicative risk model predicts the combined CE of multiple predators by summing the independent effects of predators on instantaneous mortality rates (i.e., summing the independent effects of predators on log-transformed estimates of percent survival) when these assumptions are true (Wilbur and Fauth 1990; Billick and Case 1994; Sih et al. 1998). In other words, the multiplicative risk model predicts the combined mortality risk imposed by multiple predators (μ_na_) on their prey when the predators do not alter the mortality risk imposed by each other as:


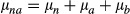
Model(1)

where μ_*n*_ is the instantaneous mortality risk (i.e., CE) imposed by newts, μ_*a*_ is the instantaneous mortality risk (i.e., CE) imposed by *Anax*, and μ_*b*_ is the background instantaneous mortality risk of prey in the absence of predators. Model 1 can be modified to predict the combined mortality risk imposed by multiple predators (μ_na_) on their prey when predators modify the mortality risk of prey to other predators:


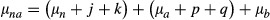
Model(2)

where *j* refers to the NCE that nonphysical interactions with *Anax* has on the instantaneous mortality risk imposed by newts, *k* refers to the NCE that physical interactions with *Anax* (e.g., interference competition or intraguild predation) has on the instantaneous mortality risk imposed by newts, *p* refers to the NCE that nonphysical interactions with newts has on the instantaneous mortality risk imposed by *Anax*, and *q* refers to the NCE that physical interactions with newts has on the instantaneous mortality risk imposed by *Anax*. Our mechanistic model 2 is similar to a mechanistic model described by Rudolf (2008), however, we employ an additive approach to modifying mortality risk rather than a multiplicative approach (i.e., μ_*n*_ + *j* + *k* rather than μ_*n*_*jk*).

We present model 2 with an additive approach because we present a statistical approach below that allows us to efficiently estimate all the parameter estimates (and their standard errors) in model 2, evaluate whether each parameter is significantly different than zero, and compare the observed combined mortality risk to those expected from several null models that make different assumptions about *j* and *k* while only conducting a single analysis of variance (ANOVA) with planned contrasts. Furthermore, because *j*, *k*, *p*, and *q* refer to the extent to which one predator alters the CE of another predator via a NCE, the influence of CE and NCE is expressed in similar units using the additive approach which allows us to directly evaluate how much each contributes to the combined effect of multiple predators on prey mortality.

In our study, we assume that *k* = *q* = 0 because (1) intraguild predation is unlikely between these two predator species and (2) it is not possible to experimentally separate the influences of *j* and *k* from the influences of *p* and *q* (although we can estimate *j* and *p*, we cannot estimate *k* and *q*) when the removal of any potential behavioral influence by one predator likely also removes the influence of physical interference (although *k* and *q* could be estimated for intraguild predation if appropriate treatments that manipulate the densities of each predator to reflect the influence of intraguild predation are conducted). Nonetheless, our design can suggest the importance of *k* and *q* if our models, which assume they are zero, fail to adequately predict the combined effect of multiple predators.

We tested eight hypotheses regarding the CE and NCE of multiple predators on a common prey. The first four hypotheses (contrasts 1–4) evaluated whether the CE of each predator species (i.e., μ_*n*_ and μ_*a*_) and the NCE of each predator species on the CE of the other predator species (i.e., *j* and *p*) are different from zero. The last four hypotheses (contrasts 5–8) evaluated whether the observed prey mortality risk in the presence of multiple predators is different from that expected by (i) model 2 when *j* = *p* = 0, (ii) model 2 when *j* = 0 and *p* reflects the observed effect on μ_*a*_, (iii) model 2 when *j* reflects the observed effect on μ_*n*_ and *p* = 0, and (iv) model 2 when *j* reflects the observed effect on μ_*n*_ and *p* reflects the observed effect on μ_*a*_. We compared the observed prey mortality risk in the presence of multiple predator species to each of these four models to identify the model with the parameter estimates that best predicts the combined effect of multiple predators. To test these hypotheses we performed eight planned contrasts in conjunction with a one-way ANOVA comparing instantaneous mortality rates among all six treatments using PROC MIXED in SAS (Cary, NC) (Table 1). A block effect was excluded from the ANOVA model because it accounted for little variation in the data.

**Table 1:** Planned contrasts to test eight hypotheses pertaining to the impact of predators on the instantaneous mortality rates of *Bufo terrestris*

Contrast	CNLA	LA	LALN	LN	CALN	None
	(μ_*a*_, *p*, μ_*b*_)	(μ_*a*_, μ_*b*_)	(μ_*n*_, *j*, μ_*a*_, *p*, μ_*b*_)	(μ_*n*_, μ_*b*_)	(μ_*n*_, *j*, μ_*b*_)	(μ_*b*_)
1. Does *Anax* affect the mortality rate of *B. terrestris* relative to background mortality rates?H_o_ = LA − None = (μ_a_ + μ_b_) − (μ_b_) = μ_a_ = 0	0	+1	0	0	0	−1
2. Do newts affect the mortality rate *of B. terrestris* relative to background mortality rates*?*H_o_ = LN − None = (μ_n_ + μ_b_) − (μ_b_) = μ_n_ = 0	0	0	0	+1	0	−1
3. Does the nonconsumptive effect of *Anax* alter the consumptive effect of newts on *B. terrestris?*H_o_ = CALN − LN = (μ_n_ + *j* + μ_b_) − (μ_n_ + μ_b_) = *j* = 0	0	0	0	−1	+1	0
4. Does the nonconsumptive effect of newts alter the consumptive effect of *Anax* on *B. terrestris?*H_o_ = CNLA−LA = (μ_a_ + *p* + μ_b_) − (μ_a_ + μ_b_) = *p* = 0	+1	−1	0	0	0	0
5. Does model 2 adequately predict the combined effect of multiple predators when we assume that the NCE of each predator on the CE of the other predator is unimportant (i.e., *j* = *p* = 0)? This is thetraditional test of model 1.H_o_ = (LALN + None) − (LA + LN) = ((μ_n_ + 0 + μ_a_ + 0 + μ_b_) + μ_b_) − ((μ_a_ + μ_b)_ + (μ_n_ + μ_b_)) = 0	0	−1	+1	−1	0	+1
6. Does model 2 adequately predict the combined effect of multiple predators when we assume that NCE produced by nonphysical interactions with *Anax* is important (i.e., *j* ≠ 0) while the NCE produced by nonphysical interactions with newts is unimportant (i.e., *p* = 0)?H_o_ = (LALN + None) − (LA + CALN) = ((μ_n_ + *j* + μ_a_ + 0 + μ_b_) +μ_b_) − ((μ_a_ + μ_b_) + (μ_n_ + *j* + μ_b_)) = 0	0	−1	+1	0	−1	+1
7. Does model 2 adequately predict the combined effect of multiple predators when we assume that NCE produced by nonphysical interactions with newts is important (i.e., *p* ≠ 0) while the NCE produced by nonphysical interactions with *Anax* is unimportant (i.e., *j* = 0)?H_o_ = (LALN + None) − (CNLA + LN) = ((μ_n_ + 0 + μ_a_ + *p* + μ_b_) + μ_b_) − ((μ_a_ + *p* + μ_b_) + (μ_n_ + μ_b_)) = 0	−1	0	+1	−1	0	+1
8. Does model 2 adequately predict the combined effect of multiple predators when we assume that the NCEs produced by nonphysical interactions with both predators are important (i.e., *p* ≠ 0 and *j* ≠ 0)?H_o_ = (LALN + None) − (CNLA + CALN) = ((μ_n_ + *j* + μ_a_ + *p* + μ_b_) + μ_b_) − ((μ_a_ + *p* + μ_b_) + (μ_n_ + *j* + μ_b_)) = 0	−1	0	+1	0	−1	+1

Treatment codes are: CNLA = caged newt and lethal Anax, LA = only lethal Anax, LALN = lethal Anax and lethal newt, LN = only lethal newt, CALN = caged Anax and lethal newt, and None = no predators. Ho refers to the null hypothesis being tested by the contrast. µa represents the mortality risk (CE) imposed by Anax, μ*n* represents the mortality risk (CE) imposed by newts, µn represents the background mortality rate in the absence of predators, j represents the extent to which the NCE of Anax alters the CE of newts, and p represents the extent to which the NCE of newts alters the CE of Anax. Parameters in parentheses under each treatment code represent those parameters which have the potential to have a non-zero value in determining the overall mortality risk in the treatment. The absence of a parameter within the parentheses under the treatment code means that the parameter has zero influence on the overall mortality risk in the treatment.

Contrasts 5–8 evaluated whether there was a statistical interaction between the effect of newt presence and the effect of *Anax* presence; the presence of a statistical interaction between these two effects on estimates of instantaneous mortality rates means that the observed combined mortality rate is different from that expected by the null model (Sih et al. 1998; Vance-Chalcraft et al. 2004). Contrasts 5–8 differed from each other by changing the treatments used in the contrast to represent the effects of newt presence and the effect of *Anax* presence, but all four contrasts included the treatments with no predators to incorporate the influence of background mortality rates (where mortality risk is represented by μ_b_) and the treatment with lethal *Anax* and newts to provide the observed estimate for the combined effect of multiple predators (where mortality risk is represented by model 2). For example, contrast 5 includes the treatment with lethal newts alone (where mortality risk is represented by μ_*n*_ + *j* + μ_b_ and *j* = 0 because no *Anax* are present) and the treatment with lethal *Anax* alone (where mortality risk is represented by μ_a_ + *p*+ μ_b_ and *k* = 0 because no newts are present) in order to evaluate the null model outlined in hypothesis 5 – this is the traditional analysis used to evaluate model 1 or model 2 where it is assumed that *j* = *k* = *p* = *q* = 0. Contrast 6, on the other hand, includes the treatment with lethal newts and caged *Anax* (where mortality risk is represented by μ_*n*_ + *j* + μ_b_ and *j* represents the actual NCE of caged *Anax* on the CE of newts) and the treatment with lethal *Anax* alone (where mortality risk is represented by μ_*a*_ + *p* + μ_*b*_ and *p* = 0 because no newts are present) in order to evaluate the null model outlined in hypothesis 6.

Rejecting hypothesis 5 means that at least some of the NCE of predators on the CE of other predators (i.e., *j*, *k*, *p*, and/or *q*) are important parameters to include in the model, whereas failing to reject it suggests that they are unimportant. Failing to reject one of the hypotheses 6–8 indicates that we have identified a model that includes an important NCE produced by nonphysical interactions (either *j* and/or *p*) that is sufficient to predict the combined effect of multiple predators. Rejecting hypotheses 5–8 indicates that NCE produced by physical interactions (either *k* and/or *q*) are important parameters to include in the model even though it may not be possible to estimate them. We used four LSMESTIMATE statements employing the same treatment weights as the first four contrasts in PROC MIXED to obtain the least square estimates (and standard error) of μ_n_, μ_a_, *j*, and *p*. An additional LSMESTIMATE statement estimated μ_b_ by assigning a weight of zero to all treatments except for the predator-free control which received a weight of one. Predicted effects for each of the models were derived by summing parameter values assumed (although many are calculated) for each model.

There are several advantages to the statistical approach we employ in comparison to other studies which employ a mechanistic approach to evaluate how other predators alter the mortality risk imposed by other predators (e.g., Crumrine and Crowley 2003; Rudolf 2008; Crumrine 2010). First, our approach utilizes all the data together in a single analysis (rather than multiple analyses with different subsets of the data) which enhances statistical power for all hypotheses because the greater number of independent replicates in the analysis reduces the estimate of the error MS used to test hypotheses. Second, by conducting a single analysis with the data there is no variation in the error structure for different hypothesis tests that would otherwise occur if one performed multiple analyses that utilized different, but partially overlapping data sets. Third, our approach reduces the potential for committing type I errors given that fewer hypothesis tests are actually being performed. Fourth, all the parameter estimates in the mechanistic model are directly estimated from a single statistical mode rather than performing lots of analyses on different subsets of the data. Indeed, a great advantage of our approach is that it directly relates least square estimates from our statistical analysis to the parameter estimates in our mechanistic model and so there is no disconnect between the mechanistic model and the statistical model. The connection between the statistical and mechanistic model is important because our statistical model can lead to more accurate parameter estimates by allowing us to account for other sources of variation that may influence mortality risk. For example, we could use body size estimates for individual predators in each experimental unit as a covariate in the statistical analysis so that the least square parameter estimates are adjusted to account for differences in mortality risk due to differences in predator size. Fifth, the approach we outline does not require one to match individual replicates from different treatments together in order to estimate parameters or predicted mortality risks which has been a concern in other studies. Sixth, our approach allows us to evaluate whether observed mortality risk in the presence of multiple predators is statistically different from multiple null models without biasing the error structure of the analysis. Others have treated different null models as different treatments in an analysis and considered the predicted values for each treatment as independent which biases the estimate of the error MS used to test the hypotheses because the different predictions in each treatment are not really independent. Although our contrasts are not necessarily independent of each other (and we can correct for this with the false discovery rate), the data in the analysis are completely independent.

We report probability values for each contrast that were adjusted to control the false discovery rate (P_fdr_; Verhoeven et al. 2005) (See Appendix S3 for original ANOVA results) as the contrasts are not orthogonal. We present unadjusted *P*-values in Appendix S3. All statistical analyses were conducted in SAS Enterprise Guide (SAS 2010).

### Experiment 2

We quantified *Bufo* behavior in response to the NCE of: (1) a caged adult newt, (2) a caged larval *Anax*, and (3) the absence of predators in plastic tubs (34.4 × 21.4 × 11.5 cm) filled with 4 L of filtered water. Fifteen newly hatched (Gosner stages 23–28) *Bufo* tadpoles were present in all tubs. Predators were the same size as those in experiment 1. Twelve washed sweet gum leaves collected from natural ponds were placed in each tub to add structural complexity. The cages used in this experiment were the same cages used in experiment 1. Each treatment was replicated once within each of six spatial blocks. The experiment began on 22 May 2009 and ran for 4 h (∼1800–2200). We used a scan sampling technique (Altmann 1974) at hourly intervals during the experiment to facilitate the calculation of two metrics of *Bufo* behavior: (1) activity levels: the proportion of tadpoles that were either actively swimming or actively feeding in the water and (2) refuge use: the proportion of tadpoles that were hiding under the surface of the leaf litter (Appendix S2). We used SAS to conduct repeated measures ANOVA to evaluate the effects of treatments and blocks on both of these behavioral responses. We used the Ryan-Einot-Gabriel-Welsch (REGW) procedure to compare treatment means during each observation period as it is one of the most powerful pairwise comparison procedures (Day and Quinn 1989).

## Results

**Figure 1:**
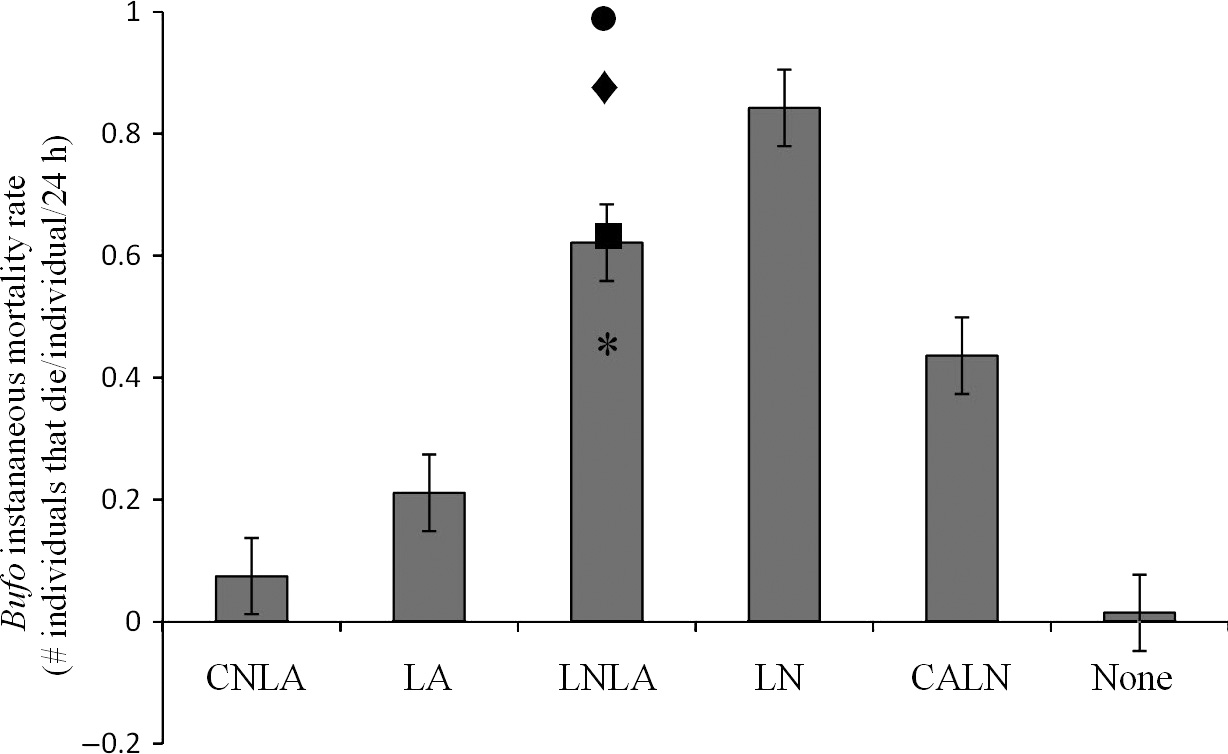
Least square mean (±1 SE) mortality rate (# individuals that die/individual/24 h) of *Bufo* in treatments varying in the occurrence of each of two predator species (*Anax* or newts). Treatment codes are as follows: CNLA, caged newt and lethal *Anax*; LA, only lethal *Anax*; LALN, lethal *Anax* and lethal newt; LN, only lethal newt; CALN, caged *Anax* and lethal newt; None, no predators. The symbol • represents the expected mortality rate of *Bufo* (1.04) for contrast 5. The symbol ▪ represents the expected mortality of *Bufo* (0.63) for contrast 6. The symbol ♦ represents the expected mortality of *Bufo* (0.90) for contrast 7. The symbol * represents the expected mortality rate of *Bufo* (0.49) for contrast 8. *N* = 7 in all cases.

No *Anax* or newts died during this study; even when placed together in the same tub. The mortality rate of *Bufo* differed among treatments in the first experiment (*F*_5, 36_ = 26.90, *P* < 0.001; Figure 1). *Bufo* mortality rates were greater in the presence of a lethal *Anax* (*F*_136_ = 4.93, *P*_fdr_ = 0.052) and in the presence of a lethal newt (*F*_136_ = 87.06, *P*_fdr_ < 0.001) than when predators were absent (Fig. 1). Mortality rates (±1 SE) were low in the absence of predators (μ_b_ = 0.0145 ± 0.0627) and not significantly different than zero (*t*_36_ = 0.23. *P* = 0.8181). The addition of a caged *Anax* caused the mortality rate of *Bufo* in the presence of a free-swimming newt to be lower than that observed if the caged *Anax* was absent (*F*_136_ = 20.94, *P*_fdr_ = 0.002). The addition of a caged newt did not cause the mortality rate of *Bufo* in the presence of a free-swimming *Anax* to differ from that observed when the caged newt was absent (*F*_136_ = 2.38, *P*_fdr_ = 0.176). Parameter estimates (and their standard errors) for μ_n_, μ_a_, *j*, and *p* are 0.8275 ± 0.0887, 0.1970 ± 0.0887, −0.4058 ± 0.0887, and −0.1368 ± 0.0887, respectively.

The observed mortality rate of *Bufo* in the presence of both predators was lower than expected when model 2 was employed where we assumed (i) the NCE of one predator on the other predator (i.e., *j* and *p*) is 0 (contrast 5: *F*_136_ = 11.09, *P*_fdr_ = 0.004), or (ii) the NCE of *Anax* on the CE of newts (i.e., *j*) is 0, but the NCE of newts on the CE of *Anax* (i.e., *p*) reflects the measured effect (contrast 7: *F*_136_ = 5.02, *P*_fdr_ = 0.052). When we assumed that the NCE of both predators was important (i.e., neither *j* nor *q* = 0), the observed combined impact of multiple predators was not statistically different from that expected by the null model (contrast 8: *F*_136_ = 0.99, *P*_fdr_ = 0.373), but the model underestimated the combined impact (0.496 vs. 0.621). The observed combined impact of multiple predators was also not statistically different from the null model where we assumed the NCE of newts on the CE of *Anax* was not important (*p* = 0), but the NCE of *Anax* on newts (i.e., *j*) reflects that the measured effect was important (contrast 6: *F*_136_ = 0.01, *P*_fdr_ = 0.92) and only slightly overestimated the combined effect (0.633 vs. 0.621).

**Figure 2:**
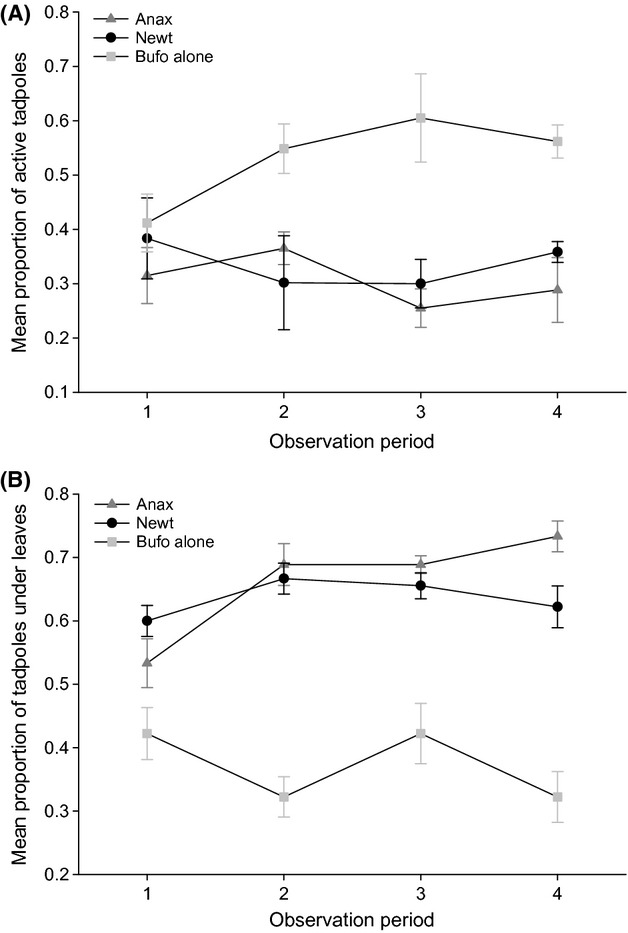
Mean (±1 SE) (A) activity levels of *Bufo* (proportion of tadpoles that were active) and (B) refuge use of *Bufo* (proportion of *Bufo* tadpoles hiding under leaf litter) in the absence and presence of one of two caged predator species (*Anax* or newts). Observation periods correspond to the number of hours after the experiment was initiated. *N* = 6 in all cases.

*Bufo* foraging activity differed among treatments in the second experiment (F_2, 15_ = 13.95, *P* < 0.001), but there was a tendency for treatment effects to vary with observation period (F_6, 45_ = 1.95, *P* = 0.094; Fig. 2A). REGW pairwise comparisons reveal that *Bufo* foraging activity was initially comparable among treatments (*P* > 0.05), but *Bufo* became less active in the presence of either predator by the second observation period (*P* < 0.05) even though foraging activity was similar in the presence of either predator at all times (*P* > 0.05). *Bufo* also differed in their microhabitat use among treatments (F_2, 15_ = 99.41, *P* < 0.001) and these differences increased with time (F_6, 45_ = 4.47, *P* = 0.001; Fig. 2B). REGW pairwise comparisons indicate that a greater proportion of *Bufo* were present in the litter refuge when predators were present during all observation periods, but there was no statistically significant difference in refuge use between the two treatments where predators were present until 4 h of exposure to predators (Fig. 2B). When differences among predators did appear, a greater proportion of *Bufo* were present in the refuge when *Anax* was present than when newts were present.

## Discussion

Predicting the combined CE of multiple predators on their prey can be difficult, especially if predators alter each other's interactions with their prey. This has been the point of studies documenting emergent MPE and these studies often report mechanisms which could explain the emergent MPE. An important unanswered question, however, is whether the reported mechanism is adequate to allow us to better predict the combined CE of multiple predators. Our study identified a mechanism to explain the occurrence of an emergent MPE, and then demonstrated that we can better predict the combined CE of multiple predators on their prey when we parameterize a multiplicative risk model with estimates of the CE of each predator species that reflect the influence of this mechanism. Although a model which included the NCE of each predator species on the CE of the other predator species enhanced our ability to predict the combined effect of multiple predator species, a model that assumed one of the NCEs was unimportant (i.e., *k* = 0) was much better. The fact that the NCE of *Anax* caused the CE (CE = μ_n_ = 0.8275 ± 0.0887; NCE = *j*−0.4058 ± 0.0887) of newts to be reduced by nearly 50% emphasizes that the NCE of predators is very important.

The mechanism that we identified to produce an emergent MPE is that the NCE of *Anax* on *Bufo* behavior altered the CE of newts on *Bufo*. Specifically, nonphysical interactions associated with the presence of *Anax*, but not newts, caused more *Bufo* to hide under leaf litter (Fig. 2B). This is supported by evidence from our second experiment, where the mean proportion of *Bufo* tadpoles hidden under leaves is greater with a caged *Anax* than with a caged newt near the end of the experiment. It appears that *Bufo* increased their usage of refuge through time when *Anax* was present, whereas *Bufo* appeared to be consistent in their refuge use through time when newts were present (Fig. 2A). As newts typically forage in the water column rather than under litter (Petranka 1998; Chalcraft and Resetarits 2003a), this behavioral response of *Bufo* to *Anax* was associated with a reduction in foraging efficiency of newts on *Bufo*. Our model, which incorporated estimates of the CE of one predator species and the nonphysical and nonconsumptive influence of another predator species would not have been able to predict the combined CE of multiple predators if the primary reason for our documented emergent MPE stemmed from physical interactions among predators (e.g., interference competition) and/or their prey (e.g., chasing prey into a refuge that is effective against both predators).

Recently, McCoy et al. (2012) argued that the detection of an emergent MPE by employing the multiplicative risk model could be misleading because most predators have a type II functional response and the multiplicative risk model assumes a type I functional response. We do not believe that the emergent MPE that we observed is misleading for at least two reasons. First, McCoy et al. (2012) reported that any bias produced from the multiplicative risk model and an additive experimental design when predators with a type II functional response are manipulated occur in the form of risk enhancement (i.e., the observed mortality of prey in the presence of multiple predators is greater than expected if the predators are foraging independently of each other). We employed an additive experimental design and observed risk reduction (the observed mortality of prey in the presence of multiple predators is less than expected if the predators are foraging independently of each other). Consequently, we did not observe the bias expected by McCoy et al.'s (2012) computer simulations. Second, we would not expect to be able to predict the combined impact of multiple predators if we did not account for the type II functional response of predators. Although we were unable to predict the combined impact of multiple predators on their prey when we did not account for the NCE of predators, we were able to predict the combined impact when we accounted for the NCE of predators. Consequently, we believe that the multiplicative risk model was adequate for predicting the combined impact of multiple predators on their prey if we account for the NCE of predators but not necessarily the functional response of predators.

Why is the fact that an emergent MPE was produced primarily by NCEs that derived from nonphysical interactions rather than physical interactions important? The production of an emergent MPE caused by nonphysical interactions with a predator indicates that predators can have an important effect on prey survival without prey having to directly encounter a predator. This point has been made before in studies arguing that NCEs of predators can have important consequences for food webs (Englund 1997; Werner and Peacor 2003; Preisser et al. 2005; Peckarsky et al. 2008; Preisser and Bolnick 2008), but most MPE studies have not explicitly addressed if NCE of predators is sufficient to explain the occurrence of an emergent MPE. Instead, many MPE studies (e.g., Soluk and Collins 1988; Sih et al. 1998; Eklöv and Werner 2000; Siddon and Witman 2004; Vance-Chalcraft et al. 2004; Vance-Chalcraft and Soluk 2005) have observed changes in prey or predator foraging behavior and implied that these changes explain an emergent MPE without actually predicting the combined effect of the predators on prey survival. Although emergent MPEs may be produced via a number of possible mechanisms, our results indicate that, at least for this particular system, knowing how species respond to the threat of predation, without even encountering the predator, is sufficient to predict the combined CE of multiple predator species on their prey.

The emergent MPE documented in our study was the result of one predator species exerting a stronger NCE on the behavior of a prey species in the food web than a second predator species. This finding suggests that the occurrence of an emergent MPE depends on the particular combination of predators present in a food web. Specifically, not all predators may exert a NCE on the behaviors of other species in the food web that is sufficiently strong to alter the CE of other species in the food web. Others have noted that the strength of NCE on prey can vary with characteristics of predators. For example, Preisser et al. (2007) found that sit-and-wait predators elicit stronger NCEs on prey than actively foraging predators which is consistent with observations during our study. *Anax* elicited a greater behavioral response in *Bufo* and is a sit-and-wait predator (Bergelson 1985; Stav et al. 2007), whereas newts are an active foraging predator (Chalcraft and Resetarits 2003b; Hunsinger et al. 2005). We believe that the change in the CE of newts occurred as a result of the addition of a caged *Anax* rather than an increase in the density of predators because we did not observe a change in the CE of *Anax* when a caged newt was added. If changes in the CE of predators were driven by changes in predator density rather than predator identity we would have expected that the addition of any caged predator would have altered the CE of a predator.

We demonstrated that the combined CE of multiple predator species on prey in our study can be better predicted when predictions are based on consumption rates of one individual predator species measured in the presence of another nonconsumptive predator species than when our predictions are based on consumption rates of each individual, free-swimming predator species alone. Most importantly, our results indicate that the primary mechanism through which an emergent MPE arises in the system we studied did not require any physical interaction between predators or between predators and prey where death is not a result for the prey. One potentially broad implication of our study is that the occurrence of a predator species in a system could compromise the efficiency of keystone predators to capture their prey without ever encountering keystone predators or their prey. Newts are known to function as keystone predators because they selectively consume competitively superior larval anuran species (like *Bufo*) which increases the relative abundance of competitively inferior larval anuran species (Morin 1983; Wilbur 1987; Chalcraft and Resetarits 2003a). Previous work in this system has shown that another predator species, *Siren intermedia*, has the ability to facilitate the keystone ability of newts by increasing the range of prey densities over which newts can alter the outcome of competitive interactions among larval anurans (Fauth and Resetarits 1991). Our study points to how another common predator species, *Anax* spp., can reduce the keystone ability of newts by reducing the efficacy in which newts can consume a competitively dominant larval anuran. If the ability of a species (such as newts) to function as a keystone predator is compromised (i.e., become a “bald arch” to follow the engineering term that refers a degraded keystone), then cascading effects could alter biodiversity of the local community. Further investigation is needed to understand the interplay between emergent MPEs and NCEs and how this influences prey biodiversity.

## Supporting Information

**Appendix S1.** Studies evaluating the contribution of NCEs of predators to emergent MPEs.

**Appendix S2.** Supplementary methods.

**Appendix S3.** Original ANOVA results from planned contrasts testing the eight hypotheses pertaining to the impact of predators on the instantaneous mortality rates of *Bufo terrestris. P*-values have not been adjusted. The degrees of freedom for contrasts are 136 in all cases. H_*o*_ refers to the null hypothesis being tested by the contrast. µ_*a*_ represents the mortality risk (CE) imposed by *Anax*, µ_*n*_ represents the mortality risk (CE) imposed by newts, µ_*n*_ represents the background mortality rate in the absence of predators, *j* represents the extent to which the NCE of *Anax* alters the CE of newts, and *p* represents the extent to which the NCE of newts alters the CE of *Anax*.
