# Supplementary material for: Nonconsumptive effects in a multiple predator system reduce the foraging efficiency of a keystone predator
Source: Ecol Evol. 2013 Aug 1;3(9):3063–72. doi: 10.1002/ece3.691 (PMC3790551; doi:10.1002/ece3.691)
Supplement: Supplementary file 1 [file ece30003-3063-SD1.docx]

Appendix S1. Studies evaluating the contribution of NCEs of predators to emergent MPEs.

Studies that have partitioned different mechanisms through which an emergent MPE is produced have found that behavioural responses to the presence of predators account for the majority of the emergent MPE (Crumrine & Crowley 2003; Griffen & Byers 2006, Rudolf 2008). All of these studies focused on systems with intraguild predation, however, and Griffen & Byers (2006) lacked supporting behavioural observations. Furthermore, due to the nature of the manipulations in these studies, none of these studies were able to determine whether the non-consumptive effects were due to physical interactions among the species or non-physical interactions.

Work by Relyea & Yurewicz (2002) indicates that changes in the behaviour of species in response to non-physical interactions among species present in the food web can have an important effect on species interactions; however, their study did not provide an explicit test for an emergent MPE with the multiplicative risk model. Their study applied a square root transformation to survival data prior to analyses which resulted in a test of a different (i.e., neither the additive nor multiplicative risk model) and undescribed biological model for predicting the combined effect of multiple predator species on their prey (Billick & Case 1994; Wootton 1994).

**Literature Cited for Appendix S1**

Billick, I. & Case, T.J. (1994) Higher order interactions in ecological communities:

what are they and how can they be detected? *Ecology*, 75, 1529-1543.

Crumrine, P.W. & Crowley, P.H. (2003) Partitioning components of risk reduction in a

dragonfly-fish intraguild predation system. *Ecology*, 84, 1588-1597.

Griffen, B.D. & Byers, J.E. (2006) Partitioning mechanisms of predator interference in

different habitats. *Oecologia*, 146, 608-614.

Relyea, R.A. & Yurewicz, K.L. (2002) Predicting outcomes from pairwise interactions:

integrating density- and trait-mediated effects. *Oecologia*, 131, 569-579.

Rudolf, V.H.W. (2008) The impact of cannibalism in the prey on predator-prey systems.

*Ecology*, 89, 3116-3127.

Wootton, J.T. (1994) Putting the pieces together: testing the independence of interactions

among organisms. *Ecology*, 75, 1544-1551.
