# Supplementary material for: Nonconsumptive effects in a multiple predator system reduce the foraging efficiency of a keystone predator
Source: Ecol Evol. 2013 Aug 1;3(9):3063–72. doi: 10.1002/ece3.691 (PMC3790551; doi:10.1002/ece3.691)
Supplement: Supplementary file 2 [file ece30003-3063-SD2.doc]

Appendix S2. Supplementary methods

*Collection sites and dates*

*Bufo terrestris* egg masses were collected for both experiments at the West Research Campus of East Carolina University on 9 May 2009. Late instar larval *Anax longipes* (*Anax* in the main text) and adult newts were collected on 13-14 May 2009 in the Croatan National Forest near New Bern, North Carolina. *Bufo* tadpoles began hatching 12 May 2009. We counted and randomly assigned newly hatched tadpoles to experimental units at abundances indicated in the main text.

*Experiment 1*

Blocks were defined as a spatial cluster of tubs that were studied at a particular time. We could not initiate all replicates at the same time due to limited availability of tubs so we initiated replicates of each treatment on three different days ( n=3 on each of May 15 and 17, 2009, and n=1 on May 19, 2009). Tubs were cleaned (10% bleach solution) before initiating another replicate in the same tub to erase any remnants of the previous community.

*Experiment 2*

For each of the four observation periods, we observed each tub by scan sampling for 15 seconds and counted 1) the number of *Bufo* tadpoles that were swimming in the water or were actively feeding with tails undulating, 2) the number of *Bufo* tadpoles that were present in the water column, 3) the number of *Bufo* tadpoles that were on the surface of the leaf litter, and 4) the number of *Bufo* tadpoles that were under the surface of the leaf litter. All tadpoles survived in this behavioural experiment so any tadpoles not observed in the water column or visible on the surface of the litter were determined to be under the litter.

Supplementary Table S1. Density of predator and prey species found in nature.

| Species | Density estimates (individuals/m^2^) | Reference |
| --- | --- | --- |
| *Bufo* spp. | 67.9-998.2 | Alford 1999 |
| *Anax* spp. | up to 8 | Van Buskirk 1988 |
| *Anax* spp. | 0.5-16 | Chalcraft unpublished data |
| *Notophthalmus viridescens* | 1.7-15.5 | Morin 1983 |

**Literature Cited for Appendix S2**

Alford, R.A. (1999) Ecology: resource use, competition and predation. *Tadpoles: The Biology of Anuran Larvae,* (eds R.W.

McDiarmid & R. Altig), pp. 240-278. University of Chicago Press, Chicago, IL, USA.

Morin, P.J. (1983) Predation, competition, and the composition of larval anuran guilds.

*Ecological Monographs*, 53, 119-138.

Van Buskirk, J. (1988) Interactive effects of dragonfly predation in experimental pond

communities. *Ecology*, 69, 857-867.
