# Supplementary material for: Nonconsumptive effects in a multiple predator system reduce the foraging efficiency of a keystone predator
Source: Ecol Evol. 2013 Aug 1;3(9):3063–72. doi: 10.1002/ece3.691 (PMC3790551; doi:10.1002/ece3.691)
Supplement: Supplementary file 3 [file ece30003-3063-SD3.doc]

Appendix S3. Original ANOVA results from planned contrasts testing the eight hypotheses pertaining to the impact of predators on the instantaneous mortality rates of *Bufo terrestris.* P-values have not been adjusted. The degrees of freedom for contrasts are 1,36 in all cases. H_o_ refers to the null hypothesis being tested by the contrast. µ_a_ represents the mortality risk (CE) imposed by *Anax*, µ_n_ represents the mortality risk (CE) imposed by newts, µ_n_ represents the background mortality rate in the absence of predators, j represents the extent to which the NCE of *Anax* alters the CE of newts, and p represents the extent to which the NCE of newts alters the CE of *Anax*.

| Contrast |  |  |
| --- | --- | --- |
|  | F-value | p-value |
| 1. Does *Anax* affect the mortality rate of *B. terrestris* relative to background mortality rates?  H_o_=LA-None=( µ_a+_ µ_b_)-( µ_b_)=µ_a_=0 | 4.93 | 0.033 |
| 2. Do newts affect the mortality rate *of B. terrestris* relative to background mortality rates*?*  H_o_= LN-None=(µ_n+_ µ_b_)-(µ_b_)=µ_n_=0 | 87.06 | <0.001 |
| 3. Does the non-consumptive effect of *Anax* alter the consumptive effect of newts on *B. terrestris?* H_o_=CALN-LN=( µ_n_+j+ µ_b_)-( µ_n+_µ_b_)= j=0 | 20.94 | <0.001 |
| 4. Does the non-consumptive effect of newts alter the consumptive effect of *Anax* on *B. terrestris?*  H_o_= CNLA-LA=( µ_a_+p+ µ_b_)-( µ_a+_µ_b_)=p=0 | 2.38 | 0.132 |
| 5. Does model 2 adequately predict the combined effect of multiple predators when we assume that  the NCE of each predator on the CE of the other predator is unimportant (i.e., j=p=0)? This is the  traditional test of model (1).  H_o_= (LALN+None)-(LA+LN)=(( µ_n_+ 0+µ_a_+0+µ_b_)+µ_b_)-((µ_a_+ µ_b)_+(µ_n_+µ_b_))=0 | 11.09 | 0.002 |
| 6. Does model 2 adequately predict the combined effect of multiple predators when we assume that  NCE produced by non-physical interactions with *Anax* is important (i.e., j≠0) while the NCE  produced by non-physical interactions with newts is unimportant (i.e., p=0)?  H_o_= (LALN+None)-(LA+CALN)=(( µ_n_+ j+µ_a_+0+µ_b_)+µ_b_)-((µ_a_+ µ_b_)+(µ_n_+j+µ_b_))=0 | 0.01 | 0.925 |
| 7. Does model 2 adequately predict the combined effect of multiple predators when we assume that  NCE produced by non-physical interactions with newts is important (i.e., p≠0) while the NCE  produced by non-physical interactions with *Anax* is unimportant (i.e., j=0)?  H_o_= (LALN+None)-(CNLA+LN)=(( µ_n_+ 0+µ_a_+p+µ_b_)+µ_b_)-((µ_a_+p+µ_b_)+(µ_n_ +µ_b_))=0 | 5.02 | 0.031 |
| 8. Does model 2 adequately predict the combined effect of multiple predators when we assume  that the NCEs produced by non-physical interactions with both predators are important (i.e., p≠0 and  j≠0)?  H_o_=(LALN+None)-(CNLA+CALN)=(( µ_n_+ j+µ_a_+p+µ_b_)+µ_b_)-((µ_a_+p+µ_b_)+(µ_n_ +j+µ_b_))=0 | 0.99 | 0.326 |
